# Personal

**Published:** 1907-08

**Authors:** 


### PERSONAL.

Dr. and Mrs. M. D. Mann, of Buffalo, recently sailed for Europe to spend the remainder of the summer in a vacation tour.

Dr. William C. Krauss, of Buffalo, is spending his vacation in Europe. He left Buffalo July 24, and will return to take up his professional work during the first week in October. He will make Munich his central visiting place.

Dr. L. S. McMurtry and daughter, Miss Marie-Louise, of Louisville, sailed for Europe July 18, 1907, in the S. S. Amerika. They will be absent two months engaged in continental travel.

Dr. and Mrs. Joseph M. Mathews, of Louisville, are spending the summer in Europe, traveling in company with Dr. and Miss McMurtry.

Dr. G. A. Himmelsbach, of Buffalo, is traveling in Europe for recreation to which he will devote the remainder of the summer.

Dr. A. Vander Veer, of Albany, delivered the oration in surgery at the 141st annual meeting of the Medical Society of the State of New Jersey, June 25-27, 1907. He chose for his subject, "Some Observations on Four Decades of American Surgery—1866 to 1906." His discourse was replete with interesting data showing medical progress during these forty years.

Dr. Edwin Walker, of Evansville, Ind., was tendered a complimentary banquet by his professional colleagues on Wednesday evening, July 3, 1907, in commemoration of his election as first vice-president of the American Medical Association. Seventy physicians were present, Dr. L. Worsham presiding as toastmaster.

